# An artificial bee colony optimization algorithms for solving fuzzy capacitated logistic distribution center problem

**DOI:** 10.1016/j.mex.2024.102964

**Published:** 2024-09-19

**Authors:** Yasser M. Ayid, Mohammad Zakaraia, Mohamed Meselhy Eltoukhy

**Affiliations:** a Mathematics Department, Faculty of Sciences and Arts, Al-Kamil University of Jeddah, Saudi Arabia; b Operations Research Department, Faculty of Graduate Studies for Statistical Research, Cairo University, Egypt; c Department of Information Technology, College of Computing and Information Technology at Khulais, University of Jeddah, Jeddah, Saudi Arabia

**Keywords:** Artificial bee colony optimization, Fuzzy sets, Capacitated logistic distribution center, Design of experiments, An Artificial Bee Colony Optimization Algorithm for Solving Fuzzy Capacitated Logistic Distribution Center Problem

## Abstract

This paper presents a methodological approach to solving the fuzzy capacitated logistic distribution center problem, with a focus on the optimal selection of distribution centers to meet the demands of multiple plants. The distribution centers are characterized by fixed costs and capacities, while plant demands are modeled using fuzzy triangular membership functions. The problem is mathematically formulated by converting fuzzy demands into crisp values, providing a structured framework for addressing uncertainty in logistic planning. To support future research and facilitate comparative analysis, 20 benchmark problems were generated, filling a gap in the existing literature. Three distinct artificial bee colony algorithm variants were hybridized with a heuristic: one using the best solution per iteration, another incorporating chaotic mapping and adaptive procedures, and the third employing convergence and diversity archives. An experimental design based on Taguchi's orthogonal arrays was employed for optimizing the algorithm parameters, ensuring systematic exploration of the solution space. The developed methods offer a comprehensive toolkit for addressing complex, uncertain demands in logistic distribution, with code provided for reproducibility.

Key contributions include:

• Development of a fuzzy model for the selection of distribution centers with fixed costs and capacities under uncertain plant demands.
• Generation of 20 benchmark problems to advance research in the fuzzy capacitated logistic distribution center problem domain.
• Integration of a heuristic approach with three distinct ABC algorithm variants, each contributing unique methodological insights.

## Graphical abstract

**Figure:**
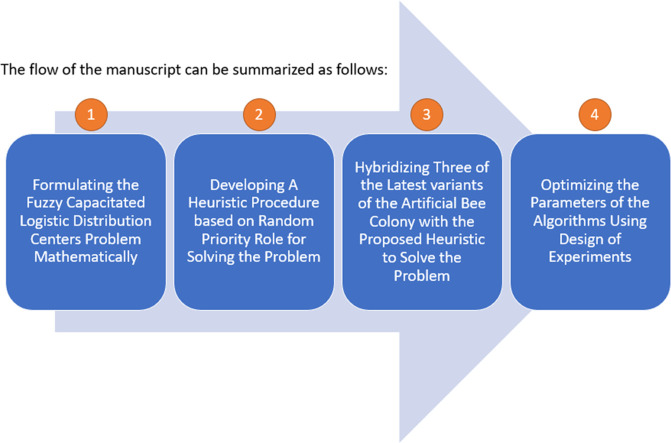

Specifications table

**Table:** 

Subject area:	Computer Science
More specific subject area:	*Metaheuristics and Fuzzy sets*
Name of your method:	*An Artificial Bee Colony Optimization Algorithm for Solving Fuzzy Capacitated Logistic Distribution Center Problem*
Name and reference of original method:	*Jiang, C., Yang, S., Nie, P., & Xiang, X. (2023). Multi-objective structural profile optimization of ships based on improved Artificial Bee Colony Algorithm and structural component library. Ocean Engineering, 283.*https://doi.org/10.1016/j.oceaneng.2023.115124 *Karaboga, D., & Gorkemli, B. (2014). A quick artificial bee colony (qABC) algorithm and its performance on optimization problems. Applied Soft Computing Journal, 23, 227–238.*https://doi.org/10.1016/j.asoc.2014.06.035 *Ye, T., Wang, H., Zeng, T., Omran, M. G. H., Wang, F., Cui, Z., & Zhao, J. (2024). An improved two-archive artificial bee colony algorithm for many-objective optimization. Expert Systems with Applications, 236.*https://doi.org/10.1016/j.eswa.2023.121281
Resource availability:	https://github.com/MZakaraia/FCLDCP/

## Background

The logistics and supply chain management field has increasingly recognized the importance of incorporating uncertainty into decision-making processes, particularly in the selection and operation of distribution centers. Distribution centers play a critical role in ensuring that products are efficiently stored, processed, and distributed to meet the demands of various plants or end-users. Traditional models often assume that demands and costs are precisely known; however, in real-world scenarios, these parameters are frequently uncertain and may vary due to a range of factors, including market fluctuations, supply chain disruptions, and unpredictable consumer behavior. Fuzzy logic provides a robust framework for modeling such uncertainties by representing uncertain parameters with membership functions rather than fixed values. Specifically, fuzzy triangular membership functions have been widely adopted due to their simplicity and effectiveness in capturing uncertainty in demand. By integrating fuzzy logic into the capacitated distribution center selection problem, it is possible to develop more realistic models that better reflect the complexities of real-world logistics. However, solving fuzzy capacitated logistic distribution center problems (FCLDCP) presents significant challenges, particularly in the absence of established benchmark problems that can be used for validation and comparison. This gap in the literature necessitates the creation of standardized problem instances that can serve as a foundation for future research.

Optimization algorithms, particularly those based on swarm intelligence, have shown great promise in solving complex, combinatorial optimization problems like the one at hand. The Artificial Bee Colony algorithm (ABC), inspired by the foraging behavior of honey bees, is a relatively recent addition to the family of swarm intelligence algorithms. It has been successfully applied to various optimization problems due to its simplicity, flexibility, and ability to escape local optima. Recent research has produced several ABC variants, each incorporating different mechanisms to enhance performance, such as chaotic mapping, adaptive procedures, and diversity preservation strategies. Given the complexity of the fuzzy capacitated logistic distribution center problem, hybrid approaches that combine heuristic methods with these advanced ABC variants are particularly well-suited for exploring the solution space effectively. Such hybridization allows for the strengths of different algorithms to be leveraged, potentially leading to more robust and high-quality solutions.

This paper contributes to the field by addressing the fuzzy capacitated logistic distribution center problem using a novel methodological approach. It formulates the problem mathematically, generates new benchmark problems, and integrates a heuristic with multiple ABC variants to explore their performance in this context. The use of Taguchi's orthogonal arrays for parameter optimization further enhances the rigor and reproducibility of the proposed methods, paving the way for future research and application in this critical area of logistics and supply chain management.

## Method details

The methods in this paper start with the mathematical formulation of the fuzzy logistic distribution center problem. After reviewing recent advancements in artificial bee colony algorithms, three variants are selected and hybridized with a proposed heuristic to tailor them for solving the problem. To support this, benchmark problems are generated, and a design of experiments is conducted to optimize the algorithm parameters. The optimized settings are then applied to the algorithms, and convergence speed curves are plotted to determine the most effective algorithm for solving the problem.

## Mathematical formulation

The main objective in the capacitated logistic distribution center problem is to find the minimum total cost of transportation and operations. The problem under investigation in this paper discusses the problem under uncertainty by having fuzzy demand constraints, where the demands are represented by fuzzy triangular numbers. Each demand in the problem has three values, minimum, most likely, and maximum. The mathematical model of the problem can be formulated as follows:

Notations:

**Table:** 

$C_{ij}$	Transportation cost from the distribution center ito plant j
$x_{ij}$	The amount transferred from distribution center ito plant j
$f_{i}$	The fixed cost related to utilizing the distribution center i
$y_{i}$	A binary decision variable ensures the selection of the distribution center i, where $y_{i}=1$ when the distribution center iis utilized, and $y_{i}=0$ if it is not utilized
${\tilde{D}}_{j}$	The fuzzy demand of plant j
$Q_{i}$	The capacity of the distribution center i

The mathematical model:

(1) $$Z=Min.\;\sum_{i=1}^{n}\sum_{j=1}^{m}C_{ij}x_{ij}+\sum_{i}^{n}f_{i}y_{i}$$

*S.t.*

(2) $$\sum_{j=1}^{m}x_{ij}\leq \;Q_{i}y_{i},\;\forall i=1,\cdots ,\;n$$

(3) $$\sum_{i=1}^{n}x_{ij}\in {\tilde{D}}_{j},\;\forall j=1,\cdots ,\;m$$

(4) $$x_{ij}\geq 0\;and\;y_{i}\in \left\{0,\;1\right\}$$

**Fig. 1:**
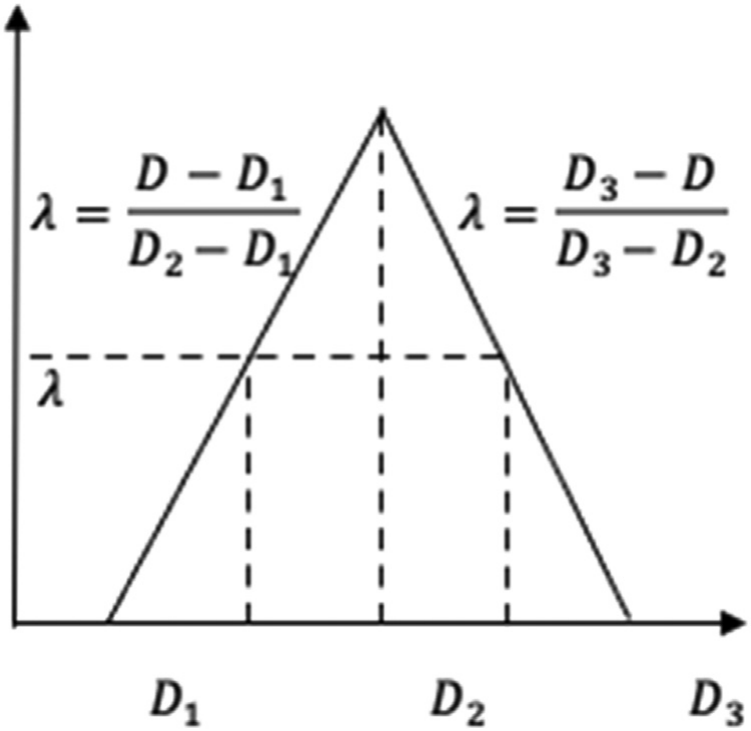
Demand fuzzy triangular membership function.

The objective function (1) seeks to find the minimum total cost of both transportation and operating costs of the selected logistic distribution centers. The set of constraints (2) is the capacity constraints of the selected logistic distribution centers. The fuzzy demand constraints are formulated in the set of constrains (3). As previously mentioned, the fuzzy numbers ${\tilde{D}}_{j}$ is a fuzzy triangular number that has three values, minimum, most likely, and maximum. They are collected under uncertainty based on experts’ impression about the expected demand. As known, the fuzzy triangular membership function has two sides, one for linear increasing and another for linear decreasing. These two sides of the fuzzy triangular membership function led to the conversion of each fuzzy demand constraint into two constraints: one for the increasing side and another for the decreasing side. According to Fig. 1 the value of *Ď_j_* can be converted to $D_{j}$ at a specific λusing two equations, either the equation that represents the linear increasing or the equation represents the linear decreasing of the fuzzy triangular membership function. Eq. (5) shows the value of $D_{j}\;$in case of the linear increasing side of the fuzzy triangular membership function and Eq. (6) shows the calculation of $D_{j}\;$in case of the linear decreasing side. Notice that each $D_{j}\;$has three values $D_{1j\;}$, $D_{2j}$, and $D_{3j}$ that represent the minimum, most likely, and maximum values of the demand j.

(5) $$D_{j}=\lambda \left(D_{2j}-D_{1j}\right)+D_{1}$$

(6) $$D_{j}=D_{3j}-\lambda \left(D_{3j}-D_{2j}\right)$$

Now, the set of constraints (3) can be converted to two sets of constraints using the Eqs. (5) and (6).

(7) $$\sum_{i=1}^{n}x_{ij}\geq \lambda \left(D_{2j}-D_{1j}\right)+D_{1j},\;\forall j=1,\cdots ,\;m$$

(8) $$\sum_{i=1}^{n}x_{ij}\leq D_{3j}-\lambda \left(D_{3j}-D_{2j}\right),\;\forall j=1,\cdots ,\;m$$

The new mathematical model after adding the set of constraints (7) and (8) can be reformulated as follows:

(9) $$\min Z=\sum_{i=1}^{n}\sum_{j=1}^{m}C_{ij}x_{ij}+\sum_{i}^{n}f_{i}y_{i}$$

*S.t.*

(10) $$\sum_{j=1}^{m}x_{ij}\leq \;Q_{i}y_{i},\;\forall i=1,\cdots ,\;n$$

(11) $$\sum_{i=1}^{n}x_{ij}\geq \lambda \left(D_{2j}-D_{1j}\right)+D_{1j},\;\forall j=1,\cdots ,\;m$$

(12) $$\sum_{i=1}^{n}x_{ij}\leq D_{3j}-\lambda \left(D_{3j}-D_{2j}\right),\;\forall j=1,\cdots ,\;m$$

(13) $$x_{ij}\geq 0\;and\;y_{i}\in \left\{0,\;1\right\}$$

The membership value λ in the new mathematical model is to be determined by the decision maker to reflect his tolerance and satisfaction level.

## Previous work on artificial bee colony optimization

This section covers some of the latest research work presented in artificial bee colony optimization algorithm. Ozcan and Simsir [1] proposed a replacement scheduling model for a rail production line, integrating the Bin Packing algorithm and Artificial Bee Colony algorithm. Sahin et al. [2] developed two combinatorial Artificial Bee Colony algorithms for generating test suites in object-oriented software, improving coverage and efficiency by introducing an archive. Li et al. [3] developed a discrete artificial bee colony optimization algorithm for solving the traveling salesman problem. They used local enhancement strategy to escape from local optima and 2-opt strategy with fixed neighborhood search that uses swapping pairs of cities. Li et al. [4] combined the fuzzy conjunctive maps with an artificial bee colony to optimize the prediction of a time series prediction model. Anuradha et al. [5] proposed a sentiment analysis method using a modified artificial bee colony algorithm for feature selection. Chai et al. [6] addressed challenges in Fetal Electrocardiogram extraction. They combined least square adaptive filter with a discreate artificial bee colony algorithm to minimize noise and maximize signal quality. Li et al. [7] proposed a discrete artificial bee colony for the flexible flow shop scheduling problem. Their proposed algorithm changes the number of employed bees dynamically according to the solutions quality. Zhu et al. [8] developed a binary artificial bee colony and implemented to solve the un-capacitated facility location problem and the maximum cut problem. Jin et al. [9] proposed an improved artificial bee colony that covers a new representation scheme and population method for solving the remanufacturing system scheduling problem. Mumtaz et al. [10] formulated a mixed-integer linear programming model for assembly line balancing and scheduling of automated guided vehicles of the customized printed circuit board. They developed a hybrid algorithm that incorporates genetic algorithm and the artificial bee colony to solve this problem. Kolukisa et al. [11] proposed a logistic regression trained by a parallel artificial bee colony with hyper-parameter optimization for increasing the detection of the network intrusions.

Ren et al. [12] proposed a mathematical model for a bi-objective disassembly line scheduling problem. The optimized objectives in their paper are to minimize the disassembly time and the smoothing index. Their proposed algorithm for solving this problem is an artificial bee colony with Q-learning. Liao et al. [13] tackled a flexible job shop scheduling problem with extra resource constraints. They formulated a mixed-integer linear programming model for the problem and solved it using artificial bee colony. Cui et al. [14] studied the multi-robot path planning in continuous known environment. Their objective is to find the optimal collision-free paths. They developed an artificial bee colony to solve the problem. Ni et al. [15] proposed a multiple strategy Q-learning integrated with the artificial bee colony. They implemented the algorithm to solve the unmanned vehicle path planning problem. Ye et al. [16] developed an artificial bee colony that is based on two archive method, convergence and diversity archives. These two archives lead to three different search strategies. Jiang et al. [17] proposed a chaotic mapping for improving the nectar sources of the artificial bee colony and adaptive search for leading and following bees, respectively. They solved a multi-objective structural profile of ships problem. Table 1 in the supplementary materials shows the literature summary. Yang et al. [18] proposed a fault diagnosis algorithm, which combined an improved artificial bee colony (ABC) algorithm with K-means clustering to address issues in diagnosing pile machinery faults.

**Table 1:** Literature summary.

Author	Year	Application	Modification
Karaboga and Basturk [19]	2007	Solving multi-variable functions	Standard version of ABC
Yang et al. [18]	2023	Diagnosing pile machinery faults	Hybridization with the K-mean clustering
Ozcan and Simsir [1]	2009	Preventive maintenance with replacement scheduling in continuous production lines	Hybridization with the Bin Packing algorithm
Sahin et al. [2]	2021	Whole test suite generation	Archive-based multi-criteria Artificial Bee Colony algorithm
Jiang et al. [17]	2023	A multi-objective structural profile of ships problem	A chaotic mapping for improving the nectar sources
			Adaptive search for leading and following bees
Li et al. [3]	2024	Traveling salesman problem	Discrete ABC
			local enhancement strategy
			2-opt strategy with fixed neighbourhood
Li et al. [4]	2024	Prediction of a time series prediction model	Fuzzy conjunctive maps with ABC
Anuradha et al. [5]	2024	Feature selection	A sentiment analysis method using ABC
Chai et al. [6]	2024	Fatal Electrocardiogram extraction	Discrete ABC
			least square adaptive filter with ABC
Li et al. [7]	2024	Flexible flow shop scheduling problem	Discrete ABC
			Dynamic number of employed bees
Zhu et al. [8]	2024	Un-capacitated facility location problem and maximum cut problem	Binary ABC
Jin et al. [9]	2024	Remanufacturing system scheduling problem	New representation scheme
Mumtaz et al. [10]	2024	Assembly line balancing and scheduling of automated guided vehicles	N/A
Kolukisa et al. [11]	2024	Network intrusions detection	A logistic regression trained by a parallel artificial bee colony with hyper-parameter optimization
Ren et al. [12]	2024	A bi-objective disassembly line scheduling problem	ABC with Q-learning
Liao et al. [13]	2024	A flexible job shop scheduling problem with extra resource constraints	N/A
Cui et al. [14]	2024	Multi-robot path planning in continuous known environment	N/A
Ni et al. [15]	2024	Unmanned vehicle path planning problem	Multi strategy Q-learning with ABC
Ye et al. [16]	2024	N/A	ABC with convergence and diversity archives

This literature review aims to identify three cutting-edge artificial bee colony algorithms to address the Fuzzy capacitated logistic distribution center Problem using a proposed heuristic approach. The comparative results section will then analyze the performance of these selected algorithms.

## Standard artificial bee colony algorithm

The artificial bee colony algorithm, introduced in recent years by Karaboga and Basturk [19], is a relatively new swarm intelligence algorithm. It emulates the optimization-seeking strategy found in the honey-harvesting behavior of a bee colony. In the ABC model, the colony has three bee groups: employed bees, onlookers, and scouts. Each food source is associated with a single artificial employed bee. Consequently, the number of employed bees in the colony determines the quantity of food sources surrounding the hive. Employed bees venture to their designated food sources, return to the hive, and commence searching in the corresponding area. If an employed bee's assigned food source is abandoned, it transforms into a scout, initiating a search for a fresh food source. Onlookers observe the movements of employed bees and select food sources based on their activities. As a result, the algorithm comprises three phases: the employed phase, the onlooker phase, and the scout phase.

### The employed bees phase

The employed bees phase starts in the initialization of the algorithm, which initialize the algorithm with a set of vectors that represent the nectar sources. These vectors are to be generated using Eq. (14). The LBand UB are the lower and upper bounds of the vector's components. popsizeis the population size, and D is the dimension of the vector.

(14) $$x_{ij}=LB+rand\left(0,\;1\right)\left(UB-LB\right),\;\forall i\in \left\{1,\cdots ,\;popsize\right\},\;j=1,\cdots ,\;D$$

After generating the initial population that represents the nectar sources and evaluating their fitness values according to Eq. (15), the employed bees start to search for new nectar sources by applying local search. According to this local search, a greedy selection will be made to select the new nectar sources with respect to the highest fitness values. Eq. (16) is the used equation to perform the local search. The vector $v_{i}\;$represents the new nectar source, vector $x_{i}$ represents the current nectar source, and vector $x_{k}$ is a randomly selected nectar source. The value of $\phi_{ij}$ is randomly generated from the interval [−a,a], where a plays the role of the scaling factor that determines the location of the new nectar source between the vectors $x_{i}\;$and $x_{k}$*.*

(15) $$f_{i}=\left\{ \begin{array}{c} \frac{1}{\left(1+F\left(X_{i}\right)\right)},\;F\left(X_{i}\right)\geq 0\\ 1+abc\left(F\left(X_{i}\right)\right),\;F\left(X_{i}\right)\leq 0 \end{array}\right.$$

(16) $$v_{ij}=x_{ij}+\;\phi_{ij}\left(x_{ij}-x_{kj}\right),\;\forall j=1,\cdots ,\;D$$

If $F\left(V_{i}\right)<F\left(X_{i}\right)$, then vector $V_{i}$ replaces $X_{i}$*,* and if else, then the abandonment limit of $X_{i}$ increments by 1. The best solution found (Best) is to be updated to use in the onlooker phase.

### The onlooker bees phase

The onlooker bees play a crucial role in monitoring the employed bees and identifying new nectar sources based on the optimal positions of the nectar sources in relation to their fitness values. During this phase, the randomly selected nectar source is replaced by selecting a new nectar source using a selection probability determined by Eq. (17). In this context, the new nectar sources $x_{k}$are chosen randomly from those with the highest selection probability.

(17) $$P_{i}=\frac{f_{i}}{\sum_{i=1}^{popsize}f_{i}},\;\forall i=\left\{1,\cdots ,\;Popsize\right\}$$

The randomly selected nectar $x_{k}\;$in Eq. (16) in this phase will be selected according to the highest selection probability, where the nectar source that has higher selection probability has higher chance to be used in Eq. (16).

### The scout bees phase

The scout bees are responsible for searching for new nectar sources; once a source is depleted, it gets abandoned. In the algorithm, an abandonment limit needs to be determined for each nectar source to restrict the search process associated with it. If the search process fails to improve the solutions, a new nectar source is generated using Eq. (14).

## Heuristic approach

The heuristic approach mirrors the steps of the least cost method algorithm outlined in [20,21], commonly employed for solving transportation problems. However, it incorporates a -random priority weighted vector that will be used later as the position of the bees in the proposed artificial bee colony algorithm. Before heading to summarize the steps of the heuristic approach, the demands of the problem have to be converted to their crisp values utilizing the set of constraints (11) and (12) by constructing Eq. (18).

(18) $$D_{j}=D_{1j}+r_{1}\times D_{3j}+\lambda \left(D_{3j}-D_{2j}-r_{1}\times \left(D_{2j}-D_{1j}\right)\right)$$

The proposed heuristic approach uses a modified bubble sort procedure to sort the indices of both the plants and distribution centers according to their priority weighted vectors [22]. The steps of the modified bubble sort procedure are as follows:

Algorithm 1 *BubbleSortProcedure(x, y)*

**Table:** 

$Set\;y_{length}=∥y∥$
j=0
test=1
Whiletest=1do:
test=0
$for\;i=\{1,\cdots ,\;y_{length}-j-1\}\;do:$
$z_{1}=y_{i}$
$z_{2}=x_{i}$
$y_{i}=y_{i+1}$
$x_{i}=x_{i+1}$
$y_{i+1}=z_{1}$
$x_{i+1}=z_{2}$
test=1
j=j+1
Returny

The steps of the heuristic approach for solving the problem based on priority weighted vectors for both plants and distribution centers are as follows:

Algorithm 2 *HeuristicApproach(D, Q,PlantsPriority, CentersPriority)*

**Table:** 

*1*	InitiateSolutionasm×nzerosmatrix
*2*	D=Updatethefuzzytriagulardemands ofDusingequation(18)
*3*	Plants=(1,⋯,m)
*4*	Centers=(1,⋯,n)
*5*	Plants= BubbleSortProcedure(PlantsPriority,Plants)
*6*	Centers= BubbleSortProcedure(CentersPriority,Centers)
*7*	While∥Plants∥≠ 0\ do:
*8*	P=thefirstplantinPlants
*9*	C=thefirstdistributionCenterinCenters
*10*	WhilePinPlantsdo:
*11*	$if\;D_{P}<Q_{C}\;do:$
*12*	$Solution_{P,C}=D_{P}$
*13*	$D_{P}=0,\;Q_{C}=Q_{C}-D_{P},\;and\;delete\;P\;from\;Plants$
*14*	$Else\;if\;Q_{C}<D_{P}\;do:$
*15*	$Solution_{P,C}=Q_{C}$
*16*	$Q_{C}=0,$ $D_{P}=D_{P}-\;Q_{C},\;and\;delete\;C\;from\;Centers$
*17*	Else:
*18*	$Solution_{P,C}=Q_{C}$
*19*	$Q_{C}=0,\;D_{P}=0,\;and\;delete\;both\;C\;and\;P\;from$ CentersandPlants,respectively
*20*	ReturnSolution

The Dand Qinputs of the heuristic approach are the demand and capacity vectors that are associated to the plants and distribution centers, respectively. The returned solution from the heuristic approach is to be evaluated using (9). The PlantsPriorityand CentersPriorityinputs are the priority vectors that will be used in the search procedure of the proposed artificial bee colony algorithm.

## The proposed artificial bee colony for solving FCLDCP

The proposed heuristic generates an initial random solution by leveraging the PlantsPriority and CentersPriority vectors. In the context of solving the FCLDCP, the ABC algorithm's initial population is established through the initiation of a set of random solutions employing this heuristic approach. Subsequently, the ABC algorithm generates new solutions primarily through the mutation of the PlantsPriorityand CentersPriority vectors. Eq. (14) is applied to these vectors, enabling the generation of new priorities. These newly generated priorities are then utilized in conjunction with the heuristic approach to derive solutions for the given problem. The fitness is determined by calculating the objective value of the solution obtained through the heuristic procedure and subsequently applying Eq. (15). The rest of the algorithm will then be applied until reaching the maximum number of iterations. The pseudo code of the ABC algorithm for solving FCLDCP can be summarized as follows:

Algorithm 3 *ABCAlgorithm(Popsize, MaxItr, a, abandonmentLimit)*

**Table:** 

*1*	$Set\;abandonmentVector=(A_{i}=0|i=1,\;\cdots ,\;Popsize)$
*2*	GenerateinitialPopulationPlantsPriority withPopsizenectorsourcesusingequation(14)
*3*	GenerateinitialPopulationCetersPriority withPopsizenectorsourcesusingequation(14)
*4*	Itr=1
*5*	whileItr≤MaxItrdo:
*6*	Updatenectarsourcesusingequation(16) forbothPlantsPriorityandCetersPriority foreachnectarsourceinPopulation
*7*	$F(X_{i})=$ HeuristicApproach(D,Q,PlantsPriority,CetersPriority) foreachnectarsourceinpopulation
*8*	$Calculate\;Evals=\left(f_{i}|i=1,\cdots ,\;Popsize\right)\;using\;equation\;\left(15\right)$
*9*	Best=argmin(Evals)
*10*	$Calculate\;P_{i}=\frac{f_{i}}{\sum_{i=1}^{Popsize}f_{i}},\;\forall i=\left\{1,\cdots ,\;Popsize\right\}$
*11*	Updatenectarsourcesusingequation(16) forbothPlantsPriorityandCetersPriority
*12*	UpdateabandonmentVector
*13*	Replacethenectarsourcesthatreached theabondonmentlimit
*14*	Itr=Itr+1
*15*	Returnbest

This paper presents three versions of the ABC algorithm. All the three versions are hybridized with the proposed heuristic approach like the previous pseudo code after adding their modifications. The first ABC algorithm is found in Karaboga and Gorkemli [23]. It replaces Eq. (16) by Eq. (19) in Algorithm 3.

(19) $$v_{ij}=x_{best}+\;Rand\left(0,1\right)\left(x_{best}-x_{ij}\right),\forall j=1,\cdots ,D$$

The second ABC algorithm is the one found in Jiang et al. [17]. It uses an adaptive search mechanism to balance the search between the global and local search. It utilizes an increasing scaling factor (ω) that helps in the calculations of the adaptive factor $c_{i}$ and the selection probability ($P_{i}$). The ω scaling factor can be found by Eq. (20), where a is selected to be 0.2 and b is selected to be 0.8. The algorithm also uses the degrees of goodness (μ) to calculate the adaptive factor $c_{i}$, where its value can be calculated using Eq. (21). Finally, the adaptive factor $c_{i}$ then can be calculated using Eq. (22).

(20) $$\omega =b+\left(a-b\right)\frac{Iteration}{MaxItr}$$

(21) $$\mu =\frac{rank_{i}}{Popsize}$$

(22) $$c_{i}=\sin\left(\frac{2\mu^{\omega }}{\pi }\right)$$

After calculating $c_{i}$, Eq. (16) can be modified as follows:

(23) $$v_{ij}=x_{ij}+\;c_{i}\left(x_{ij}-x_{kj}\right)+\left(1-c_{i}\right)\left(x_{best}-x_{ij}\right),\;\forall j=1,\cdots ,D$$

Eq. (23) plays the role of changing nectar source position in case that its associated evaluation is better than the current nectar source. The onlooker bees phase mainly based on utilizing a selection probability that can be calculated using Eq. (17). Jiang et al. [17] modified the selection probability equation as Eq. (24).

(24) $$P_{i}=\frac{\omega {\left(1-\omega \right)}^{rank_{i}-1}}{1-{\left(1-\omega \right)}^{Popsize}}$$

In the onlooker phase of Jiang et al. [17], the solutions that have the highest $P_{i}$ will be modified using Eq. (23). If any new modified nectar source is better than its previous position, then it will replace it. The initial population of Jiang et al. [17] proposed ABC algorithm is to be generated using chaotic mapping. In this procedure, each solution of the initial population will be generated using a chaotic random number ($\gamma_{k}$) follows Eq. (25). The value of $\gamma_{k}$ is then used to generate the $k^{th}$ nectar of the population as shown in Eq. (26). So, Eq. (26) replaces Eq. (14) in Algorithm 3.

(25) $$\left\{ \begin{array}{c} h_{0}=rand\left(0,1\right)\\ h_{k+1}=4h_{k}\left(1-h_{k}\right)\\ \gamma_{k+1}=\frac{2}{\pi }\arcsin\left(\sqrt{h_{k+1}}\right) \end{array}\right.$$

(26) $$x_{ij}=LB+\gamma_{k}\left(UB-LB\right),\;\forall i\in \left\{1,\cdots ,\;popsize\right\},\;j=1,\cdots ,\;D$$

The third version of the selected ABC algorithms for solving the FCLDCP is found in Ye et al. [16]. They integrated convergence and diversity archives in the algorithm. The convergence archive stores the non-dominated solutions, and the diversity archives stores the dominated solutions. Both the convergence and diversity archives have archive size that represents the number of solutions of each. If the archive size in the convergence archive is reached, then the worst solution of the convergence archive will be removed. In case the diversity archive reached its archive size, then the best solution on it is to be removed. The process replaces the abandonment procedure of the ABC algorithms. The convergence archive is to be updated in the employed bees- phase. The onlooker bees-phase utilizes the convergence archive to update the new nectar sources positions using a selection probability value calculated using Eq. (27).

(27) $$P_{i}=\frac{f\left(X_{i}\right)-f\left(X_{best}\right)}{f\left(X_{worst}\right)-f\left(X_{best}\right)}$$

In the scout bees-phase a dynamic probability is to be calculated using Eq. (28). This dynamic probability is used to tradeoff between selecting a solution from the convergence archive or staying in the current nectar position. The update of the diversity archive is done in this phase.

(28) $$\alpha =1-\frac{Iteration}{MaxItr}$$

The python codes of the proposed three hybridized algorithms can be found in https://github.com/MZakaraia/FCLDCP GitHub repository.

## Benchmark problems generation

Since the fuzzy capacitated logistic distribution center problem formulated in this paper is unique and not found in any other research papers, benchmark problems have been generated using Python. The Generate Problems code, available at https://github.com/MZakaraia/FCLDCP GitHub repository, generates these benchmarks. The code accepts parameters such as the number of problems, the minimum and maximum values for transportation costs, fixed costs, demands, capacities, the number of centers, and the number of plants. Demands are represented with three values: the minimum and maximum (the two least likely values) and the most likely value, using fuzzy triangular values. The generated data includes transportation costs, fixed costs for the distribution centers, demands, and capacities. For benchmarking purposes, we generated 20 problems using this code, all available at the aforementioned GitHub repository. These problems involve 30 centers and 20 plants, making them suitable for comparative analysis.

## Experimental design

**Fig. 2:**
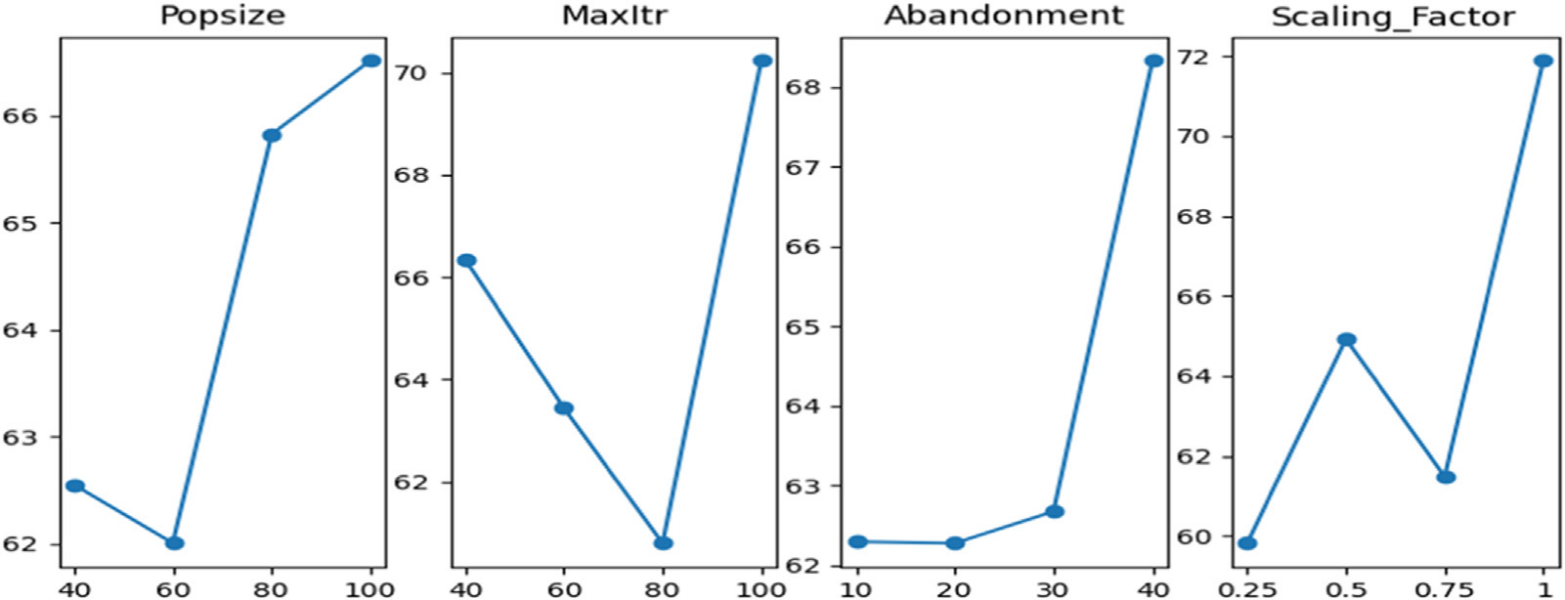
Main effects plot of S/N ratio of ABC-I.

**Fig. 3:**
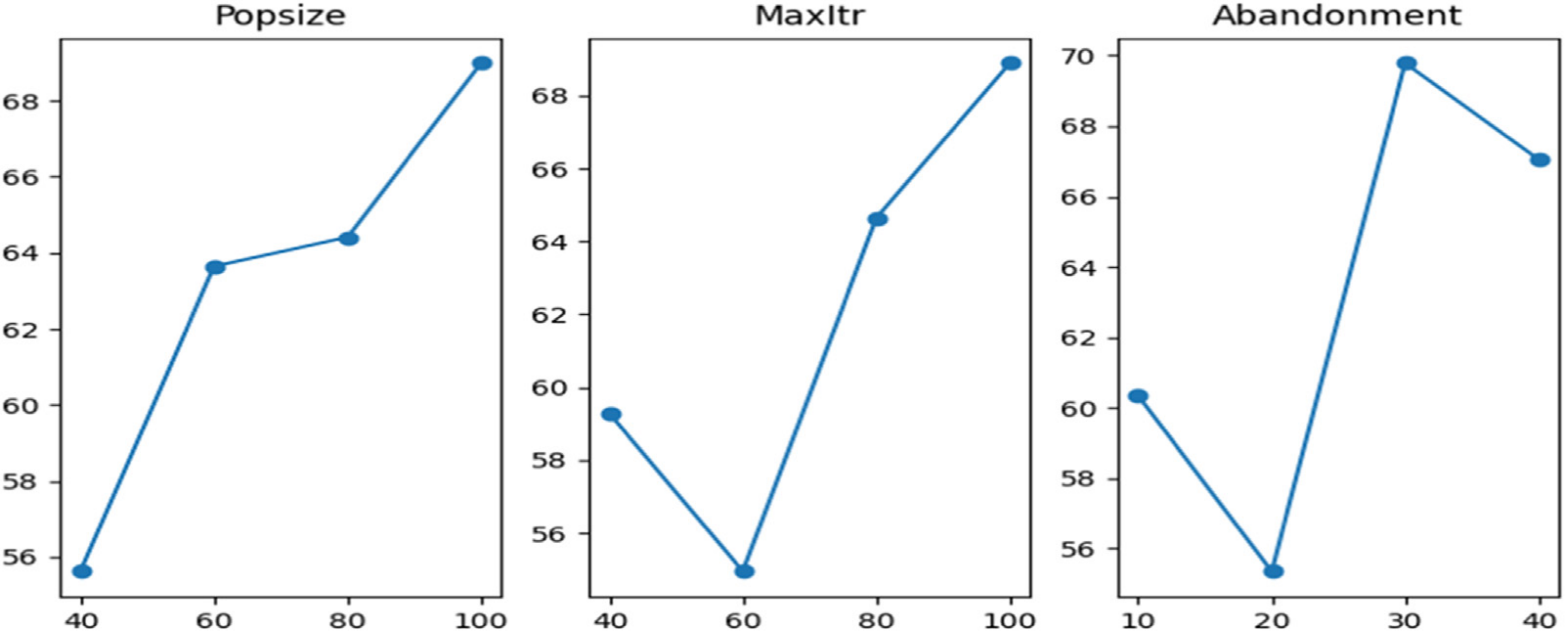
The main effects plot of S/N ratio of ABC-II.

**Fig. 4:**
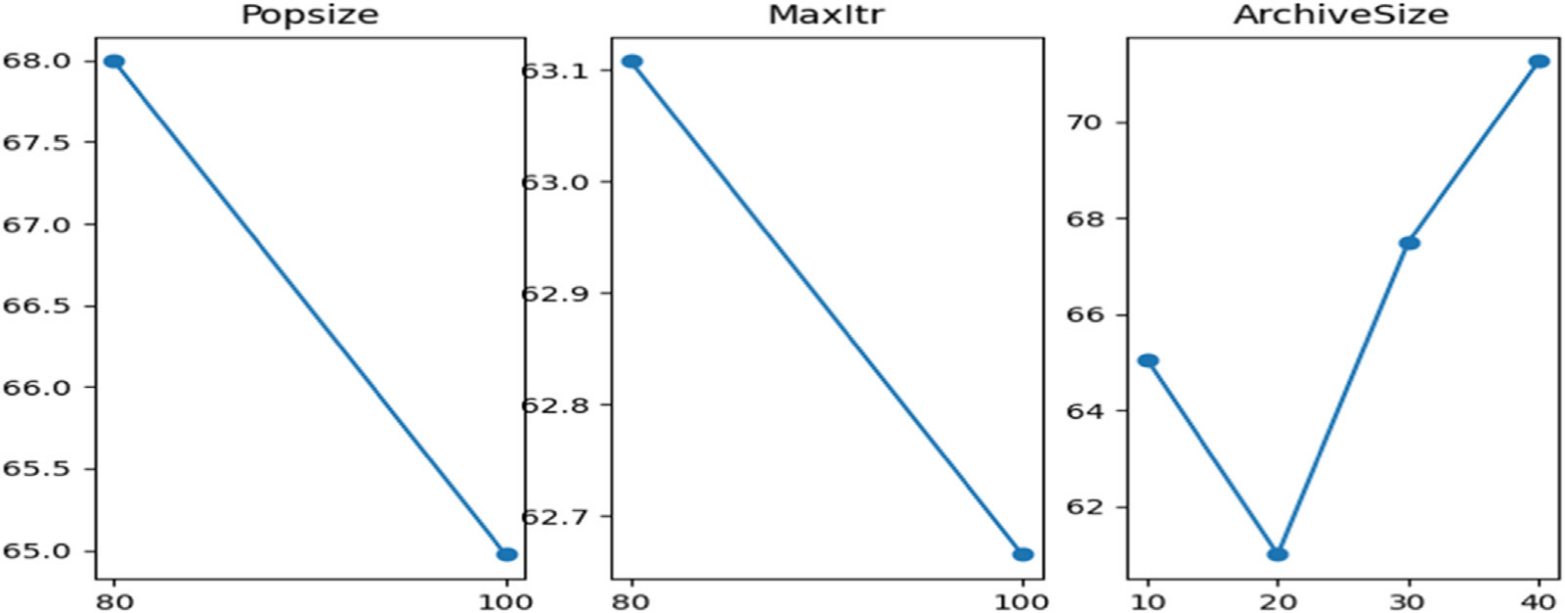
The main effects plot of S/N ratio of ABC-III.

In this section, the three proposed algorithms’ parameters are optimized using Taguchi's design of experiments [24]. The shared parameters between the three algorithms are MaxItr and Popsize. The selected parameter levels for both parameters are 40, 60, 80, and 100. For ABC-I and ABC-II the abandonment limit parameter levels are 10, 20, 30, and 40. For ABC-III the selected parameter levels for the archive size parameter are 10, 20, 30, and 40. The parameter levels of the scaling factor of ABC-I are 0.25, 0.5, 0.75, and 1. The degrees of freedom for ABC-I are 3 for the parameter levels and 1 for the overall means. So, the total degrees of freedom are 13. Therefore, the selected Taguchi's orthogonal array is $L_{16}$. For both ABC-II and ABC-III, the total degrees of freedom are 10. Hence, the selected Taguchi's orthogonal array is also $L_{16}$. The selected number of repetitions for each experiment is 5. So, the total number of experiments for each algorithm is 80 experiments. The reason of choosing to optimize the parameter levels using Taguchi's method instead of the full factorial design is that the number of experiments required for each algorithm after repetitions is $4^{4}\times \;5=1280$ experiments for ABC-I, and $4^{3}\times \;5=320$ experiments for both ABC-II and ABC-III. The signal to noise ratio is used to obtain main effects of each level that compromise between the means and standard deviation, which means the efficiency and robustness, respectively. The main effects plot of ABC-I can be found in Fig. 2. The main effects plot of ABC-II is shown in Fig. 3, and the main effects plot of ABC-III is found in Fig. 4.

The optimal parameter levels for each parameter in each algorithm can be summarized in Table 2.

**Table 2:** The optimized parameter levels for each algorithm.

Algorithm	*MaxItr*	Popsize	Abandoment	Scaling factor	Archive size
ABC-I	100	100	40	1	–
ABC-II	100	100	30	–	–
ABC-III	80	80	–	–	40

To test the convergence speed of the algorithms, the previously mentioned settings in Table 2 are used with the algorithms on the 20 benchmark problems. Fig. 5 in supplementary materials shows that ABC-I outperforms the other algorithms in terms of convergence speed, where it provides faster convergence in 13 problems of 20 benchmark problems.

**Fig. 5:**
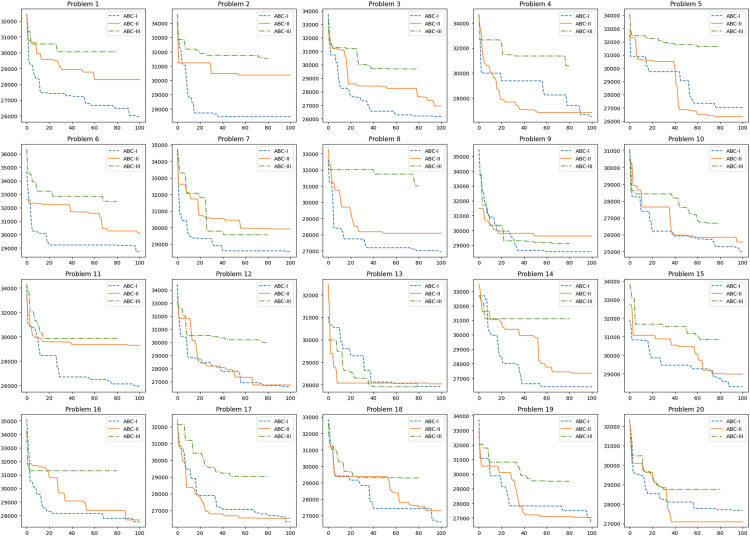
The convergence curves for the 20 problems.

## Method validation

In this section, comparative results are presented to evaluate and validate the three proposed algorithms for solving the Fuzzy Capacitated Logistic Distribution Center Problem. Each algorithm is tested using the optimal settings identified through experimental design. The same 20 benchmark problems, previously used to assess convergence speed, are utilized for these comparisons. The problems are solved under three different membership values: 0.6, 0.8, and 0.9. Tables 3, 4, and 5 in supplementary martials show the comparative results for α equal 0.6, 0.8, and 0.9, respectively. The three tables show that the ABC-I outperforms the other algorithms in terms of means, while the ABC-III shows more consistency in terms of the relative standard deviation (RSD).

**Table 3:** Comparative results for α=0.6.

**Table 4:** Comparative results for α=0.8.

**Table 5:** Comparative results for α=0.9.

## Limitations

‘Not applicable’.

## CRediT authorship contribution statement

**Yasser M. Ayid:** Investigation, Writing – review & editing, Supervision. **Mohammad Zakaraia:** Conceptualization, Methodology, Software, Visualization, Data curation, Writing – original draft. **Mohamed Meselhy Eltoukhy:** Investigation, Writing – review & editing, Supervision.

## Declaration of competing interest

The authors declare that they have no known competing financial interests or personal relationships that could have appeared to influence the work reported in this paper.

## Data Availability

All data and code links are given inside the manuscript.

## Appendix Supplementary materials

Image, application 1

Image, application 2

Image, application 3

Image, application 4

Image, application 5
